# Respiratory Dysfunction in Alzheimer’s Disease—Consequence or Underlying Cause? Applying Animal Models to the Study of Respiratory Malfunctions

**DOI:** 10.3390/ijms25042327

**Published:** 2024-02-16

**Authors:** Agnieszka Wrzesień, Kryspin Andrzejewski, Monika Jampolska, Katarzyna Kaczyńska

**Affiliations:** Department of Respiration Physiology, Mossakowski Medical Research Institute, Polish Academy of Sciences, 02-106 Warsaw, Poland; awrzesien@imdik.pan.pl (A.W.); kandrzejewski@imdik.pan.pl (K.A.); mjampolska@imdik.pan.pl (M.J.)

**Keywords:** Alzheimer’s disease, respiratory disorders, sleep obstructive apnea, animal models, hypoxia, hypercapnia

## Abstract

Alzheimer’s disease (AD) is a neurodegenerative brain disease that is the most common cause of dementia among the elderly. In addition to dementia, which is the loss of cognitive function, including thinking, remembering, and reasoning, and behavioral abilities, AD patients also experience respiratory disturbances. The most common respiratory problems observed in AD patients are pneumonia, shortness of breath, respiratory muscle weakness, and obstructive sleep apnea (OSA). The latter is considered an outcome of Alzheimer’s disease and is suggested to be a causative factor. While this narrative review addresses the bidirectional relationship between obstructive sleep apnea and Alzheimer’s disease and reports on existing studies describing the most common respiratory disorders found in patients with Alzheimer’s disease, its main purpose is to review all currently available studies using animal models of Alzheimer’s disease to study respiratory impairments. These studies on animal models of AD are few in number but are crucial for establishing mechanisms, causation, implementing potential therapies for respiratory disorders, and ultimately applying these findings to clinical practice. This review summarizes what is already known in the context of research on respiratory disorders in animal models, while pointing out directions for future research.

## 1. Introduction: Alzheimer’s Disease Neurodegeneration

Alzheimer’s disease (AD) was first described by German neuropathologist Alois Alzheimer in 1906 and named in his honor a few years later. It is a chronic, progressive neurodegenerative brain disease that causes the death of nerve cells. This disease mechanism is largely associated with the accumulation of extracellular senile plaques composed of amyloid β and the intracellular aggregation of hyperphosphorylated tau protein into neurofibrillary tangles (NFTs), leading to the loss of neuronal connections in the brain, neuronal death, and brain atrophy [1]. The cause of Alzheimer’s disease is not well understood, but there are many environmental and genetic risk factors associated with its development. Other risk factors include a history of head trauma, aging, lifestyle activities, clinical depression, high blood pressure, and sleep-disordered breathing (SDB) [2,3]. This disease process causes damage to the neocortex and hippocampus, leading to mental dementia. AD is the most common cause of dementia, accounting for more than 60% of all cases [4]. Over time, this disease completely prevents the patient from functioning properly in daily life, carrying out work, and disrupts their social interactions. In the familial form of early-onset AD, in which several genetic mutations have been identified, such as mutations in the amyloid-β precursor protein (AβPP), presenilin 1 gene, and presenilin 2 gene, the symptoms usually appear before the age of 60 and progress rapidly [5]. The majority of AD patients, more than 95% of all cases, are of the sporadic type with undetermined etiology and late onset after age 65 [6]. One of the best known genetic risk factors for the sporadic form of AD is the ApoE 4/4 genotype [7,8].

It is estimated that one in nine people aged 65 and older suffers from AD. There are an estimated 6.5 million citizens in America and more than 30 million worldwide aged 65 and older living with Alzheimer’s dementia. This number could increase significantly in the future unless there are medical breakthroughs to prevent, slow down, or treat Alzheimer’s disease [9,10]. Needless to say, caring for the growing number of patients with dementia carries enormous social and economic costs [11].

## 2. Respiratory Disturbances in AD

**Figure 1:**
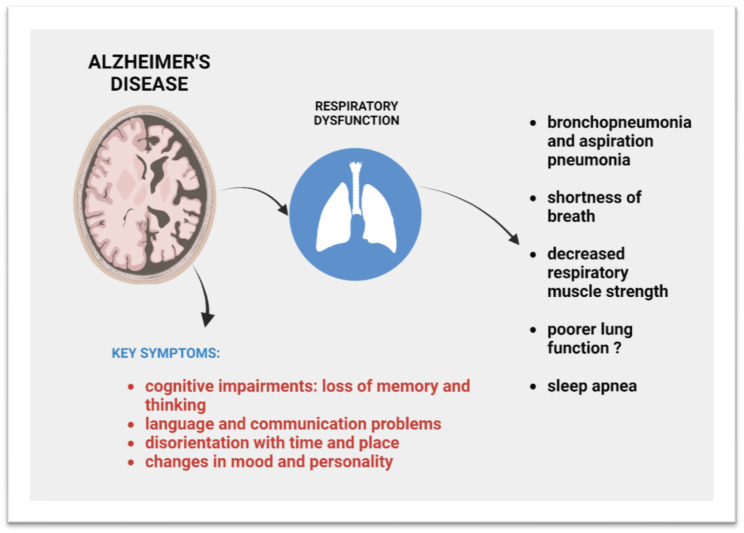
Respiratory ailments that occur in AD.

Patients with Alzheimer’s disease, in addition to well-associated cognitive impairments such as loss of memory and thinking, language problems, disorientation, mood swings, and behavioral problems, experience breathing dysfunction (Figure 1).

Respiratory diseases have been shown to be a common cause of death in patients with dementia, including those diagnosed with AD [12,13,14,15,16,17,18,19,20]. According to Brunnström and Englund [15], respiratory diseases are the cause of death in 45% of patients with dementia, compared to only 7% in the general population of a similar age. A similar result was reported in a meta-analysis and systematic review, where autopsy confirmation identified pneumonia as the cause of death in dementia patients with an outcome of 49.98% [19].

Subdividing the dementia patient population into those with Alzheimer’s disease, vascular dementia, or dementia with combined Alzheimer’s and vascular pathology, showed that respiratory disease is a more common cause of death in AD patients with a score of 55%, with bronchopneumonia (47%) and aspiration pneumonia (7%) also being the most common culprits. In the general elderly population studied for comparison, bronchopneumonia accounted for only 2.8% [15]. The high rates of death from bronchopneumonia and aspiration pneumonia in AD patients are probably associated with dysphagia and impaired coordination of swallowing and breathing, which is related to the process of human ageing; however, it seems to be exacerbated in AD [21,22,23].

Patients with AD experience a reduced ability to perform high-intensity aerobic exercise and decreased respiratory muscle strength. Even early-stage AD patients with no apparent physical deterioration, who performed a graded treadmill exercise test, showed a reduced ability to increase their breathing due to an increase in their oxygen demand compared to control adults without dementia [24]. The results indicated that AD patients may have a reduced ability to perform high-intensity aerobic exercise, which may be due to their weakened respiratory muscle strength. The decline in respiratory muscle strength was demonstrated later by measuring maximum inspiratory and expiratory pressures. Respiratory muscle strength is actually related to the aging process, but its decline was intensified in AD [25]. Spirometric tests measure lung function, including lung volumes and elastic recoil forces of the lung, and can be instrumental in assessing respiratory muscle performance. It is challenging to find studies in which these parameters have been investigated in patients with AD. Nevertheless, something may be at play, as decreased lung function, i.e., forced vital capacity (FVC) and forced expiratory volume in one second (FEV1) have been linked to weakened cognitive function and increased subcortical atrophy in middle-aged men [26]. Significant associations of lower FEV1 and FVC values with lower values of brain volume, gray matter volume, hippocampal volume, and higher volume of white matter hyperintensities have also been found in large-scale meta-analyses [27].

According to the meta-analysis by Russ et al. [28] and population-based prospective cohort study by Xiao et al. [29], people with poor lung functions have an increased risk of dementia, but what the causal relationship is between the two remains unclear. Reduced lung function can limit oxygen uptake and saturation, leading to possible hypoxia [30]. Hypoxia, meanwhile, can cause cognitive impairment and dementia, as confirmed in human and animal studies [31,32,33]. This relationship appears to be bilateral; neurodegenerative changes in the brain can adversely affect respiratory drive, respiratory regulation, and lung function, and, on the other hand, poorer lung function can cause hypoxia, which triggers neurodegenerative changes and later dementia.

Other respiratory deficits, which do not directly lead to death but impair the quality of life and lead to hypoxia, manifest as dyspnea and sleep-disordered breathing with sleep apnea predominating, which will be discussed in more detail in the following sections [34,35].

The question to be answered here is: What morphological and anatomical changes observed in AD-affected brains are responsible for respiratory dysfunction? Control of the breathing pattern is exerted primarily by areas located in the ventral respiratory group (VRG) in the ventral region of the medulla oblongata, while most AD pathology in the brainstem is distributed in a rostrodorsal direction [36]. Nevertheless, some brainstem nuclei, more or less involved in the control and modulation of breathing, and respiratory responses to hypoxia and hypercapnia have been shown to be affected by neurofibrillary tangles (NFTs) and senile plaques burden in AD patients: nucleus tractus solitarius (NTS), noradrenergic locus coeruleus, parabrachial nucleus, cholinergic pedunculopontine pars compacta (PPTg-pc) and laterodorsal tegmental nucleus (LDT), serotonergic rostral raphe complex (the dorsal raphe, paramedian, median, and linear raphe nuclei), and ambiguous nucleus innervating the muscles of soft palate, pharynx, and larynx, and dorsal motor nucleus of the vagus providing parasympathetic innervation to the bronchi and lungs [36,37]. Additionally, reticular formation nuclei such as the periaqueductal gray, the already-mentioned pontine parabrachial nuclear complex, and the intermediate reticular zone of the medulla involved in cardiovascular and respiratory control are also affected by the neurodegenerative process [36,38,39]. Another study using magnetic resonance imaging in AD subjects and control subjects also showed brainstem deformation and volume reduction in AD subjects, although it did not indicate specific nuclei of the brainstem [40]. Yet, a deficiency in the activity of the respiratory nuclei present in the brainstem, associated with neurodegenerative changes, can, in consequence, entail respiratory dysfunction.

## 3. Sleep-Disordered Breathing

Sleep-disordered breathing (SDB) encompasses a broad spectrum of sleep-related breathing disorders, including episodes of repeated respiratory arrest during sleep, in record-breaking cases up to hundreds of times per night. Apnea is considered to be the cessation of lung ventilation for more than 10 s during which blood oxygen saturation is reduced. Frequent periods of apnea lead to periods of intermittent hypoxia, hyper/hypocapnia, significant sleep fragmentation, oxidative stress, and a chronic systemic inflammatory state [41,42,43]. The most prevalent type of SDB is obstructive sleep apnea (OSA), in which the upper airway partially or completely collapses during inspiration. This collapse is partly due to reduced tension in the muscles of the upper airway, in the control of which the medullary and pontine respiratory centers are involved. The second type is central sleep apnea, characterized by unstable central ventilatory drive during sleep, in which brainstem neurons that generate respiratory rhythms transmit insufficient signals to the pharyngeal dilator muscles in the upper airway and the respiratory pump muscles of the chest wall. The third type is complex/mixed sleep apnea, which comprises a combination of obstructive and central apnea [44,45,46]. In this review, we will focus on OSA as the most common type, which, if left untreated, results in hypertension, stroke, diabetes, cardiac arrhythmias, myocardial infarction, and heart failure, as well as neurocognitive deficits in multiple domains, such as attention, memory, executive, and psychomotor function [47,48,49,50,51]. According to a 2017 systematic review covering general adult European and North American populations, the prevalence of OSA, regardless of severity, ranged from 9 to 38% [52] and was higher in men and obese individuals [52,53,54]. The prevalence of OSA considerably increases with age. For example, with the apnea–hypopnea index (determining the severity of OSA and indicating the number of complete (apnea) or incomplete (hypopnea) obstructive events per hour of sleep) of AHI ≥ 15 events/h, the incidence in the general adult population ranged from 6% to 17%, reaching 49% in advanced age [52,55]. Aging additionally increases the severity of OSA in elderly patients, even if they are physically active and do not have neuropsychiatric disorders [56].

## 4. Obstructive Sleep Apnea in Alzheimer’s Disease

Disrupted nocturnal sleep, circadian rhythm, and excessive daytime sleepiness are the core components of AD. Severe sleep disturbances are considered to be the result of damage to the neuronal pathways in patients with late-stage AD [57], as degenerative changes have been detected in sleep/wake regulatory areas such as cortical and hippocampal neurons, cortical presynaptic cholinergic nerve endings, pons, and medulla reticular formations [58]. More significantly, the sensitive areas affected by Alzheimer’s disease overlap with the brain structures affected by sleep disorders [59]. Nevertheless, it is still unclear whether sleep disorders are a cause or a consequence of AD [59].

The severity of sleep disorders in AD is associated with an increased incidence of sleep-disordered breathing, manifested as hypopnea and apnea. OSA in AD patients is believed to be a consequence of the neurodegenerative process; nevertheless, an accurate estimation of the prevalence of OSA in this population is not easy to obtain due to methodological factors such as small sample sizes, selection bias, and variability in OSA definitions and diagnostic methods. The prevalence of OSA has exceeded 40% in hospitalized patients [60], with some studies suggesting as much as 80% [34], and the severity of sleep apnea correlated with the severity of dementia in patients with AD [60,61]. A 2016 meta-analysis revealed that AD patients face a 5-fold higher risk of OSA than their age-matched controls and that approximately 50% of AD patients will face OSA after diagnosis [62]. Importantly, hypoxemia as a consequence of sleep apnea can worsen cognition and can aggravate the underlying cognitive and functional deficits inherent in AD [63,64,65].

In recent years, there has been a growing body of literature indicating that the opposite may be true and that OSA is a risk factor for the development of AD. First, sleep disorders appear many years before the clinical onset of AD. A meta-analysis involving multiple studies found that impaired breathing during sleep is associated with an increased risk of cognitive decline in older adults [66,67]. The presence of SDB was also correlated with an earlier age of cognitive decline and AD dementia [68]. According to a 40-year follow-up study that evaluated 1574 men aged 50 and older, those with sleep disturbances had a 51% higher risk of developing AD [69]. However, the limitation of self-reported sleep disorders should be mentioned here. A recently published cohort study involving patients with and without diagnosed SDB (observed until AD diagnosis, death, or the year 2015) found that patients with SDB were almost 1.58 times more likely to develop AD [70]. Remarkably, the brain changes associated with SDB in older people who do not even show cognitive impairment include greater amyloid deposition and neuronal activity in AD-sensitive brain regions [71].

Another indication that OSA may be a causal factor in AD is the observation that the treatment of obstructive sleep apnea with continuous positive airway pressure therapy (CPAP) in AD patients resulted in a moderate but sustained improvement in cognitive function or delayed cognitive deterioration [72,73,74,75,76,77].

There is growing evidence that OSA is a treatable target for limiting the progression of clinical and functional decline in patients with mild cognitive impairment (MCI) and AD [78,79,80], but further larger studies are needed to corroborate the importance of identifying and treating OSA in these patients to limit the progression of cognitive decline. The feasibility of CPAP therapy in the AD patient population, such as patient involvement, as well as assistance from the caregiver, is also an extremely important issue to consider [81].

## 5. Bidirectional Relationship between Obstructive Sleep Apnea and Alzheimer’s Disease

**Figure 2:**
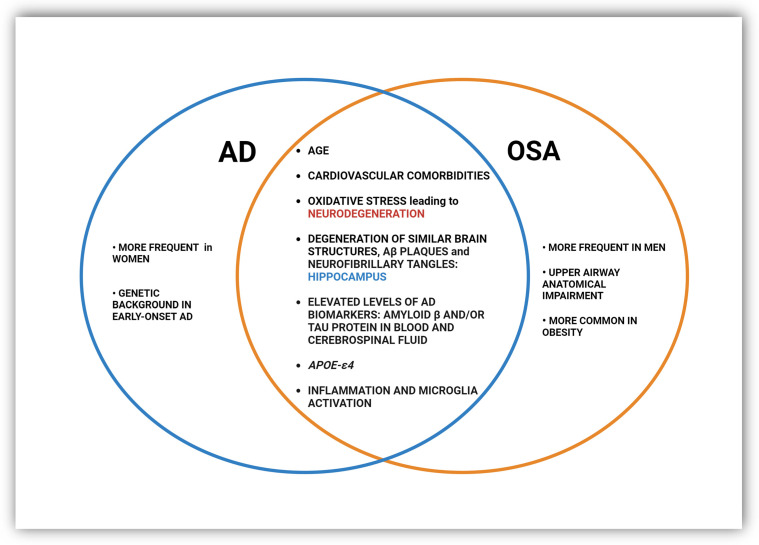
Common and distinct pathological changes and risk factors in OSA and AD.

Establishing a causal relationship between AD and OSA is not straightforward and as yet remains inconclusive. There appears to be a synergistic relationship between sleep-disordered breathing and Alzheimer’s disease because they share certain etiological and physiopathological determinants that predispose them to the development of both disorders in the same patient (Figure 2) [34,82]. First, the presence of OSA, sleep fragmentation, and nocturnal hypoxia are linked with higher cognitive impairments such as decreased memory, attention, and executive function [83,84,85,86], symptoms inherent in AD. Furthermore, it was found that the more severe the form of OSA, the stronger the association between the apnea–hypopnea index, the oxygen desaturation index, and the prediction of memory impairment [87]. The cause is oxidative stress and inflammation caused by intermittent hypoxia in sleep apnea and subsequent reoxygenation that may contribute to neuronal degeneration [42,43,88].

A recent review highlighted that nervous system inflammation and microglia activation through an inflammasome-dependent mechanism may link AD and OSA, possibly leading to a mutual and synergistic aggravation of the two diseases [3]. OSA-inherent hypoxia promotes the persistence of low-grade chronic inflammation, as evidenced by activated serum biomarkers of inflammation such as CRP, IL-6, TNF-α, NF-κB, and adhesion molecules, among many others [89,90]. Changes in cytokine levels in OSA were closely correlated with the age, body mass index (BMI), and apnea–hypopnea index (AHI) of patients [90]. A sustained inflammatory response is a constant component in the brains of AD patients, along with the presence of Aβ and NFT plaques [91,92,93,94]. This was initially thought to be a response to the neuronal loss that occurs in the disorder, but it was later revealed that inflammation can also promote and exacerbate Aβ and NFT pathologies by activating microglia, astrocytes, and other immune cells and releasing an array of pro-inflammatory and toxic products, including reactive oxygen species, nitric oxide, and cytokines [95,96].

Oxidative stress is a constant phenomenon in Alzheimer’s disease pathology [97], as increased levels of free radicals and higher levels of macromolecule oxidation have been reported in the brains of people with AD and in various experimental models [98,99,100,101]. Oxidative stress in AD is thought to be associated with the abnormal accumulation of Aβ and the deposition of neurofibrillary tangles [102]. On the other hand, OSA and intermittent hypoxia-induced oxidative stress increase tau protein levels, and their phosphorylation and leads to increased deposition of senile plaques and the formation of neurofibrillary tangles, which contribute to the pathogenesis of AD [103,104,105,106,107]. Studies in rodents showing that OSA increases the risk of AD perfectly correspond with neuronal loss caused by intermittent hypoxia, described in structures related to cognition such as the hippocampus and prefrontal cortex [108,109]. Similar hallmark pathological changes are also observed in patients with AD [3,110,111].

Interestingly, in human studies, OSA and its severity have a detrimental effect on the very same brain structures that degenerate in AD. OSA has been associated with a reduced volume of gray matter in the hippocampus, the cingulate, and the cerebellum, as well as in the temporal, frontal, and parietal lobes [112,113,114,115]. Atrophy and loss of the hippocampus and entorhinal cortex have been described in autopsy studies of brain tissue and have been correlated with the severity of OSA [116]. MRI brain scans have shown that the magnitude and frequency of oxygen desaturation in OSA are related to decreased cortical thickness in the frontal and parietal regions [117]. In this case, the relationship appears to be bidirectional; neurodegenerative changes in the AD brain may adversely affect respiratory drive, respiratory regulation, and upper airway muscle tone, and, on the other hand, OSA, by causing intermittent hypoxia, induces neurodegenerative changes and later dementia.

Another common factor is the elevated levels of AD biomarkers such as amyloid β and/or tau protein in blood and cerebrospinal fluid (CSF) observable in OSA patients, which are frequently correlated with hypoxia severity [118,119,120,121,122]. In brain tissue, however, the severity of hypoxia appeared to be a significant predictor of Aβ plaques and neurofibrillary tangles in the hippocampus but not in the brainstem [103]. More recently, attention has been drawn to the impaired glymphatic system, which removes undesired or pathological proteins from the interstitial space in the brain through exchanges between the CSF and interstitial fluid (ISF). It is during sleep that a significant increase in the flow of ISF into the CSF occurs, so the sleep fragmentation characteristic of AD and high blood pressure during OSA events contribute to insufficient clearance of tau protein and amyloid beta aggregates from the brain and CSF and their accumulation [123,124,125,126,127].

A recent study revealed a correlation of the CSF lipid profile in patients with severe OSA and mild/moderate AD with various polysomnographic measures of OSA severity. The authors proposed that increased lipoxidation in the central nervous system may be one of the mechanisms underlying the link between OSA and AD. In addition, dysregulated forms of lipids in the CSF may be potential biomarkers of OSA in AD patients [128]. Severe forms of OSA induce lipid oxidation, which, in turn, may affect APP processing by switching from a non-amyloidogenic into an amyloidogenic pathway and elevating AD pathology [129].

Shared genetic predispositions have even been proposed. The ε4 allele of the apolipoprotein E (APOE) gene remains the strongest and most prevalent genetic risk factor for AD, affecting more than half of all cases. APOE is a lipid transport protein, and lipid dysregulation has recently arisen as a key feature of AD. The mechanisms underlying the link between *APOE-ε4* and AD are far from clear, but the presence of the ε4 allele has been linked to the appearance of amyloid-β (Ab) aggregates, tau hyperphosphorylation, disorganization of mitochondrial networks, and lipid metabolism disruption [130,131,132,133]. *APOE-ε4* has also been associated with sleep abnormalities, poor sleep quality, and increased risk of insomnia [134,135]. As for apnea, on the other hand, the data are inconclusive. There are studies indicating that individuals with the ε4 allele have an increased risk for OSA, and *APOE-ε4* may predispose them to sleep apnea [136,137], while other studies did not confirm this [138,139].

## 6. Studying Respiratory Dysfunction in AD Animal Models

### 6.1. AD Animal Models

Most experimental animal models of AD are based on transgenic mice or rats with the integration of variants of human genes encoding proteins associated with AD pathology. They include mutations of genes involved in the production of amyloid plaques (APP gene, PSEN 1, PSEN 2, or a combination thereof) and neurofibrillary tangles (MAPT gene and Tau protein gene) or β-site APP-cleaving enzyme 1 (known as β-secretase). More than 200 different transgenic rodent models have been developed in Alzheimer’s disease research [140]. The most desirable models are those that show both Aβ plaques and neurofibrillary tangles. The 3×Tg mouse model widely used in AD research is considered the most complete transgenic mouse model of AD pathology [141]. However, the mutant Aβ and tau produced in their brains are not representative of sporadic AD and are otherwise highly overexpressed in a non-physiological manner. Other models rely on spontaneous changes occurring in non-human primates and dogs, which can naturally develop Aβ pathology; however, tauopathy is rare and/or limited. Another natural model is *Octodon degus*, which shows high sequence homology with human Aβ and features intracellular and extracellular Aβ accumulation, atherosclerotic plaques in old age, intracellular tau accumulation, astrocytosis, synaptic changes, and memory impairment. Despite their high similarity to human pathology in AD, the availability of this model may be limited [141]. There are also non-genetic, interventional models based on intracranial stereotactic injections of synthetic Aβ aggregates, tau-enriched AD brain lysates, and, most commonly, streptozotocin (STZ) [142].

### 6.2. Respiratory Disturbances in Streptozotocin-Induced AD Model

For unraveling the respiratory changes in Alzheimer’s disease, it is important to find a suitable animal model that demonstrates as many pathological changes in the brain resembling human AD as possible but also reflects respiratory dysfunction [143,144,145,146].

The rat model of streptozotocin is the most commonly studied in terms of respiratory changes, probably because of its simplicity, cost-effectiveness, and resemblance to the sporadic type of AD, compared to transgenic models. This model employs intracerebral injection into the lateral ventricles of a diabetogenic toxin called streptozotocin, originally identified as an antibiotic [147]. STZ disrupts insulin signaling homeostasis in the brain, leading to reduced glucose and oxygen consumption [148], according to the finding that sporadic AD is an insulin-resistant brain condition with a decrease in glucose/energy metabolism in the brain but without a systemic diabetic state [147,149]. STZ induces glial activation, neuroinflammation and oxidative stress, mitochondrial dysfunction, and tau hyperphosphorylation, and also triggers the accumulation of aggregated Aβ fragments, total tau protein, and Aβ deposits in the brain [147,150,151]. This pathological cascade leads to synaptic damage and the death of neurons, and as a consequence, adult animals develop long-term, progressive deterioration in memory, learning, and cognitive behavior. Neurochemical changes induced by intracerebroventricular (icv) STZ injections are time-dependent, and doses higher than 3 mg/kg are thought to model very aggressive neurotoxic changes. Doses of 3 mg/kg or less are regarded as adequate to mimic the slowly evolving dementing processes and symptoms of sporadic AD [152]. Most cases of AD are sporadic in origin and nature, and neuroinflammation of the brain has been identified as one of the main risk factors. Since STZ induces a similar pathology characteristic of an AD, the use of model induced by icv STZ injections appears to be a feasible experimental concept to study respiratory changes and the underlying pathophysiological mechanism in rats.

#### 6.2.1. Altered Ventilatory Response to Hypercapnia

Respiratory changes in experimental animals have so far been studied mainly in the rat STZ model of Alzheimer’s disease and by only two research groups (Table 1). Both employed STZ injections into the lateral ventricles and studied respiratory responses to hypoxia and hypercapnia. There were subtle differences in AD modeling, but primarily, the significant variations occurred in the observed breathing changes. Vincente et al. [153,154] tested respiratory responses to hypoxia and hypercapnia in Wistar rats 30 days after STZ administration (2 mg/kg), and the main effect was an increased response to hypercapnia during wakefulness. This increased sensitivity to CO_2_ has been linked to increased Aβ expression by more than 70% in the locus coeruleus (LC), a brainstem noradrenergic nucleus involved in respiratory function, precisely central chemoreception and the sensing of CO_2_ and pH [155,156,157]. The STZ treatment did not produce alterations in ventilation under room air conditions or hypoxia during sleep or wakefulness. There were no changes in tau protein phosphorylation and Aβ expression in the hippocampus or areas involved in breathing regulation: the retrotrapezoid nucleus, medullary raphe, and pre-Bötzinger/Bötzinger complex [149]. The authors also reported deficits in memory, learning, and sleep disturbances, namely increased time of wakefulness during hypercapnia.

In a later study, the authors repeated their observations, i.e., an increased sensitivity to hypercapnia in STZ-treated animals [144]. This time, Sprague Dawley rats were used, but they were given the same dose of STZ. The authors were also able to confirm the role of the LC in increased sensitivity to CO_2_ by recording its neuronal activity using the patch clamp technique and testing its response to CO_2_. It was indicated that the changes in LC neuronal output in AD rats may rather be the result of altered intrinsic properties of neurons since the number of neurons was not reduced. Enhanced responses of LC neurons to CO_2_ in the STZ model of sporadic AD may have involved STZ-induced changes in voltage-gated K+ and Na+ channels.

#### 6.2.2. Altered Ventilatory Response to Hypoxia

Ebel and coworkers [143] used awake Sprague Dawley rats, performed experiments 2 weeks after STZ injection (3 mg/kg), and displayed increased respiration at rest and reduced respiratory rate and minute ventilation responses after exposure to hypoxia. Furthermore, the sigh volume of STZ rats significantly increased during hypoxia compared to the values prior to toxin administration. The changes in the respiratory response to hypercapnia were not as apparent, and a reduced respiratory rate combined with an unchanged tidal volume did not affect the minute ventilation. Morphological alteration following icv streptozotocin injection was significant astrogliosis in the hippocampus and in the commissural part of the nucleus tractus solitarii (NTS), an area that receives afferent signals involved in cardiovascular and respiratory control such as those associated with activation of carotid body chemoreceptors [158,159,160]. The authors discussed that astrogliosis may contribute to inflammation, reactive oxygen species (ROS) formation, and prolonged glutamatergic signaling, which may impair NTS function by inducing neuronal hyperexcitability. The increase in resting minute ventilation can therefore be explained by the elevated output of respiratory centers in STZ rats, and excessive basal NTS stimulation may have an attenuating effect on the chemoreflex and the respiratory response to hypoxia.

The attenuated respiratory response to hypoxia was confirmed later [145]; this time, the animals were studied 3 weeks after STZ, and exposure to acute hypoxia (10%) lasted longer, up to 2 h. The correlation of this effect was observed with a significant decrease in c-Fos labeling as a marker of neuronal activation in the caudal/medial NTS, rostral ventral respiratory group, and Bötzinger complex, the areas that are typically activated by hypoxia. The latter was not attributed to a decrease in total cell number. The lower c-Fos staining was deemed to be due to the local impairment of neuronal energy metabolism and brain glucose dysregulation and desensitization of neuronal insulin receptors, which have been demonstrated to appear in the STZ model of AD [148,161].

Until recently, the respiratory response to hypoxia was investigated while conducting a detailed analysis of cellular and morphological changes in the NTS, as a structure involved in respiratory control and chemoreflex [146]. Two doses of STZ, 2 and 3 mg/kg, were compared, and the higher one caused more significant changes. First of all, it prompted a reduced respiratory rate response to the hypoxic stimulus, resulting in a reduced minute ventilation response. These respiratory changes were accompanied by a reduced volume of the caudal NTS in addition to hippocampal atrophy. More specific changes in the caudal NTS included reduced synaptic density with unchanged neuronal density and activation of astroglia and microglia in the caudal and intermediate regions of the NTS. All of these morphological changes in the NTS, which include the activation of microglia, may have led to neuronal hyperactivation, affecting the appropriate functioning of the chemoreflex and culminating in the blunted response to hypoxia. Administering 3 mg showed more profound changes; however, the administration of 2 mg also yielded some significant alterations, mainly in the synaptic density of the NTS and hippocampus and in the presence and morphology of astrocytes. They were also coupled with a statistically significant increase in the respiratory rate during hypoxia. The authors proposed that the latter may be a good model to study the respiratory disorders occurring in mild histopathological lesions without the still-present irreversible atrophy in the NTS [146].

Although the differences between studies in modeling AD appear to be subtle, such as different STZ doses (2 vs. 3 mg/kg), rat strains (Wistar vs. Sprague-Dawley), time frames (2 to 4 weeks after STZ), and duration of gas mixture exposure, an essential difference is that some studies observed changes in response to hypoxia [143,145,146] and others to hypercapnia [153]. This may be due to a combination of specific research conditions. For example, a lower dose and longer time after STZ administration can favor changes in the LC and an increased response to hypercapnia [153], while a higher dose may affect changes in the NTS region and alter the response to hypoxia [143,145]. The use of a specific strain may be important, as Wistar Hanover rats have been shown to be less sensitive to short-term hypoxia than Sprague Dawley rats [162]. Thus, for example, Wistar rats treated with STZ showed no change in their ventilatory response to hypoxia compared to controls [153,154] as opposed to Sprague Dawley rats, which had this response reduced [143]. Further, hypercapnia of different intensities (5% vs. 7% CO_2_) was employed in each group. A stronger stimulus, such as 7%, may recruit additional brainstem centers that are not involved in the response to 5% CO_2_, depending on their level of chemosensitivity [163]. In fact, an increased response to hypercapnia was observed in the study by Vicente et al. [153,154], which used 7% CO_2_, but no change in response to 5% hypercapnia in the study by Ebel et al. [143]. Therefore, when using the STZ model, one has to be very careful when choosing specific experimental conditions because it is possible to obtain different changes or lack thereof in response to hypoxic or hypercapnia stimuli.

The model itself appears to be valuable because even when examining the respiratory response, it demonstrates the key pathological features of AD. Briefly, the anatomical changes observed in human Alzheimer’s disease [164,165] were reflected, such as enlargement of the ventricles and reduction in the size of the hippocampus [146]. This was followed by brainstem changes in the LC, such as altered neuronal activity and increased Aβ expression [144,153], and while neuronal loss has not yet been observed here as in AD patients, where LC noradrenergic neurons are among the first to degenerate [166,167,168], this indicates that changes in this model involve the same structures as in humans. The situation is similar to the NTS, which presented caudal atrophy in a rat STZ model, accompanied by reduced synaptic density and astroglial and microglial activation in the caudal and intermediate NTS [143,145,146] and which is also affected in subjects with AD [36,169,170].

Not without meaning, intracerebroventricular administration of STZ translated into memory impairment and deficits in learning and maintenance of spatial memory in the rats studied [143,144,153]. More significantly, in the context of research into the mechanism of sleep-disordered breathing occurring in AD, this model also reproduced sleep disorders. Namely, STZ-treated rats spent more time in a state of awakeness than in NREM sleep during the exposition to normocapnia, hypercapnia, or hypoxia [153], mimicking sleep disturbances occurring in AD [32,171]. In summary, the described key pathological features of AD in the STZ model indicate its importance in translational research.

### 6.3. Respiratory Disturbances in Transgenic AD Models

#### 6.3.1. Respiratory Disturbances in the TgF344-AD Rat Model of AD

The rat model of TgF344-AD features the transgenic integration of “Swedish” mutant human APP (APPSWE) and mutant human presenilin-1 in exon Δ 9 (PS1ΔE9). This model is hallmarked by neuronal loss, amyloid plaques, tau pathology, and behavioral abnormalities that develop in an age-dependent manner [172]. Severe learning and memory impairment was observed in TgF344-AD rats at 24 months of age [172]. As the objective of the study using this model was to determine whether abnormal control of breathing develops as a prodromal trait before the onset of cognitive dysfunction, animals aged 8–11 months were studied [173]. The authors found normal breathing and ventilatory responses to hypoxia (10% in N_2_ for 10 min) and hypercapnia (5% in O_2_ for 10 min) in conscious TgF344-AD rats compared to their control animals. Also, upper airway motor function, as examined by genioglossus EMG recordings, was well preserved in transgenic rats. There was some tendency to blunt the ventilatory response to hypercapnia in TgF344-AD males and significantly shortened apnea time in response to vagus nerve stimulation with phenylbiguanide in transgenic females. The pathological changes in the brains of transgenic animals were altered APP metabolism (increased C-terminal fragments) in the pons and medulla oblongata without signs of tau hyperphosphorylation, Aβ deposition, or neuroinflammation. The authors concluded that neuronal control of respiration is preserved in TgF344-AD rats at this stage of the disease. Of all the transgenic models, this one is the only one characterized by both the presence of amyloid plaques and tau pathology. While it was studied before, there were significant pathological changes in the brain and cognitive and behavioral dysfunction. At this stage, it is difficult to talk about the usefulness of this model for studying respiratory dysfunction. Further studies on older rats are needed.

#### 6.3.2. Respiratory Disturbances in the Tau-P301L Model of AD

Tau-P301L transgenic mice gradually develop severe neuronal tauopathy in the midbrain and brainstem, with neurological symptoms beginning around 6–7 months of age, which culminates in their death at around 9–10 months of age [174]. Dutschmann et al. [175] examined the effects of brainstem tauopathy in Tau-P301L mice on breathing and motor function at a pre-symptomatic age of 3 months and at a symptomatic age of 7–8 months. Only the latter showed breathing deficits during baseline breathing, and hypercapnia manifested as the paradoxical activation of the laryngeal adductor muscles during the inspiratory phase, leading to an inspiratory airflow limitation. The latter was compensated by enlarged chest muscular work. Corresponding with the upper airway dysfunction was the detection of tau hyperphosphorylation in the brainstem nuclei involved in the control of upper airway motility, such as the Kolliker-Fuse nuclei, periaqueductal gray, and intermediate reticular nuclei. The same transgenic mice, but at a later terminal stage of the disease, showed increased upper airway dysfunction, abnormal respiratory rhythm, and abnormal respiratory regulation, with evidence of altered metabolism of serotonin and severe tauopathy in the Kolliker-Fuse nuclei, raphé obscurus, and raphé magnus [176]. Due to the lack of accumulation of amyloid plaques, Tau-P301L mice seem more suitable for modeling tauopathy than AD.

#### 6.3.3. Respiratory Disturbances in the Transgenic AβPP V717I Mouse Model of AD

A mouse model FVB-Tg (APP LD2/B6)-AβPP V717I (‘London mutation’) carrying a mutation in the amyloid β precursor protein (APP) recapitulates several histochemical, behavioral, electrophysiological, and biochemical features of AD [177]. In this mutation, altered APP processing causes an increased production of the 42-amino acid Aβ peptide, considered to be pivotal in AD pathology. This APP gene mutation and extensive AβPP overexpression have been documented in numerous familial early-onset AD cases. Transgenic mice display a gradual appearance of amyloid plaques enriched with the Aβ_42_ isoform, impaired glutamatergic signaling, decreased synaptic plasticity, cognitive dysfunction, and aggression. Remarkable and measurable Aβ pathological changes are observed at about 11–12 months of age in these mice, although behavioral alterations are present earlier than that [178]. Plaques are consistently surrounded by activated inflammatory cells, such as astrocytes and microglia, which are common in the brains of people with AD [179]. Aβ deposits in this model often form near structures immunoreactive for acetylcholinesterase, and in the cortex of aging transgenic mice, they are associated with shrinkage and deafferentation [180]. In addition, in mouse transgenic models of Alzheimer’s disease with overexpression of various APP mutations in the brain, abnormalities in glutamatergic signaling affecting learning and memory are suggested, as Aβ-induced oxidative stress deregulates the glutamatergic neurotransmission system and increases the accumulation of extracellular glutamate and activation of NMDA receptors, leading to excitotoxicity [178].

Given that APP transgenic mice exhibit many pathological features of early-onset AD, they have been used as a disease model to study respiratory responses to hypoxia and hypercapnia [181]. AD mice showed unchanged breathing during their exposure to atmospheric air, along with no difference in their ventilatory response to hypoxia, and the main finding was a significant increase in their ventilatory response to hypercapnia compared to their control group. This may indicate an impairment of the chemoreceptive respiratory nuclei in this genetic model of early AD, resulting in their increased sensitivity to CO_2_. This is consistent with the findings in STZ rats, in which increased minute ventilation was observed in response to hypercapnia [153].

Since this mouse model of AD involves the degeneration of cholinergic neurons, the authors decided to investigate whether increasing cholinergic transmission with the enzyme acetylcholinesterase inhibitor (ACh) would have any effect on respiration. Rivastigmine, given intraperitoneally, was shown to be a potent inhibitor of respiratory ventilation and hypercapnic responses, regardless of mouse genotype. A reduction in hypoxia-enhanced ventilation by rivastigmine was only observed in AD mice. The effect of rivastigmine was confirmed by reducing ACh enzyme activity in both groups equally in the hippocampus and brainstem. The authors also studied the effect of memantine, the NMDA receptor-blocking molecule used to diminish glutamate neurotoxicity and treat moderate-to-severe forms of dementia [182]; however, memantine had no significant effect on respiration [181].

Certainly, further research on transgenic disease models is needed to draw more conclusions and apply them to the study of respiratory dysfunction encountered in AD patients.

## 7. Conclusions

Respiratory disorders in Alzheimer’s disease and especially apnea have attracted considerable interest among researchers since the 1980s. So far, there has been no breakthrough in determining their underlying cause, although they worsen the quality of life, exacerbate dementia, and are one of the leading causes of death among AD patients. Recently, much attention has been paid to the causal relationship between the two diseases, namely Alzheimer’s disease and obstructive sleep apnea, the most prevalent respiratory concern in AD. This relationship encompasses such shared hallmarks between AD and OSA pathology as their co-occurrence, neurodegeneration of similar brain areas, involvement of sleep disorders, neuroinflammation, and oxidative stress. Respiratory problems, including apnea, are highly overlooked in the treatment of AD patients, and the use of continuous positive pressure, which is extremely effective in preventing apnea, can significantly improve life comfort for patients and even alleviate their cognitive impairments. Of course, the feasibility of applying the therapy to an AD patient that requires their involvement or assistance from a caregiver can be problematic; nevertheless, it is worth considering. Another notable challenge is the paucity of studies in animal models that would help clarify the causes of malfunction in breathing and enable the search for effective therapies to alleviate respiratory dysfunction. In recent years, several papers have been published examining respiratory responses to hypoxia and hypercapnia, as well as anatomical and morphological changes in the brainstem, which partly explain the altered respiratory responses in a rat model of streptozotocin-induced AD disease. There are also just a few articles on various transgenic models that have shown various respiratory changes, none of which mimic the sporadic form of AD. However, there is a lack of studies in animal models on apnea, the most common respiratory problem in Alzheimer’s disease. We therefore need further studies, including animal models, to understand the cause of the respiratory dysfunction seen in AD.

## Figures and Tables

**Table 1:** The characteristics of a rat model of streptozotocin-induced AD: respiratory responses, brain anatomical and morphological alterations, and memory and cognitive changes.

Rat Strain	STZ Dose/Time of the Test after STZ	Stimulus/Time of Exposure	Respiratory Effects	Anatomical Change	Memory and Cognitive Changes	Reference
Wistar	2 mg/kg/ 30 days	10% O_2_/60 min 7% CO_2_/60 min	↑ Response to hypercapnia	↑ Aβ expression in LC	Memory deficits and impairment in learning and retention of spatial memory (Barnes maze)	[153,154]
Sprague Dawley	2 mg/kg/~2 weeks	10% CO_2_ LC neurons tested for responses to CO_2_ (patch clamp technique)			Impairment in learning and retention of spatial memory (Moris water maze)	[144]
Sprague Dawley	3 mg/kg/ 2 weeks	8, 10, 12, and 14% O_2_/10 min 5% CO_2_/10 min	↑ Basal ventilation, ↓ response to hypoxia, and ↑ sigh volume during hypoxia	Astrogliosis in NTS and hippocampus	Memory decline (passive avoidance memory test)	[143]
Sprague Dawley	3 mg/kg/ 3 weeks	10% O_2_/2 h	↓ Response to hypoxia	↓ c-Fos in NTS, rostral VRG, Bötzinger complex, and hippocampus	Not tested	[145]
Sprague Dawley	2, 3 mg/kg/ 2–3 weeks	10% O_2_ /30 min	↓ Response to hypoxia	↑ Ventricle space, atrophy of the hippocampus and in the caudal NTS accompanied by reduction in synaptic density, and astroglial and microglial activation in the caudal and intermediate NTS	Not tested	[146]

## References

[1] Kim S., Nam Y., Kim H.S., Jung H., Jeon S.G., Hong S.B., Moon M. (2022). Alteration of Neural Pathways and Its Implications in Alzheimer’s Disease. Biomedicines.

[2] Kandimalla R., Reddy P.H. (2017). Therapeutics of Neurotransmitters in Alzheimer’s Disease. J. Alzheimer’s Dis..

[3] Ulland T.K., Ewald A.C., Knutson A.O., Marino K.M., Smith S.M.C., Watters J.J. (2021). Alzheimer’s Disease, Sleep Disordered Breathing, and Microglia: Puzzling out a Common Link. Cells.

[4] Hardy J.A., Higgins G.A. (1992). Alzheimer’s Disease: The Amyloid Cascade Hypothesis. Science.

[5] Lanoiselée H.-M., Nicolas G., Wallon D., Rovelet-Lecrux A., Lacour M., Rousseau S., Richard A.-C., Pasquier F., Rollin-Sillaire A., Martinaud O. (2017). APP, PSEN1, and PSEN2 mutations in early-onset Alzheimer disease: A genetic screening study of familial and sporadic cases. PLoS Med..

[6] Andrade-Guerrero J., Santiago-Balmaseda A., Jeronimo-Aguilar P., Vargas-Rodríguez I., Cadena-Suárez A.R., Sánchez-Garibay C., Pozo-Molina G., Méndez-Catalá C.F., Cardenas-Aguayo M.-D., Diaz-Cintra S. (2023). Alzheimer’s Disease: An Updated Overview of Its Genetics. Int. J. Mol. Sci..

[7] O’Donoghue M.C., Murphy S.E., Zamboni G., Nobre A.C., Mackay C.E. (2018). APOE genotype and cognition in healthy individuals at risk of Alzheimer’s disease: A review. Cortex.

[8] Bookheimer S., Burggren A. (2009). APOE-4 Genotype and Neurophysiological Vulnerability to Alzheimer’s and Cognitive Aging. Annu. Rev. Clin. Psychol..

[9] Haque R.U., Levey A.I. (2019). Alzheimer’s disease: A clinical perspective and future nonhuman primate research opportunities. Proc. Natl. Acad. Sci. USA.

[10] World Alzheimer Report 2022: Life after Diagnosis: Navigating Treatment, Care and Support 2022. https://www.alzint.org/resource/world-alzheimer-report-2022/.

[11] World Alzheimer Report 2022|Alzheimer’s Disease International (ADI). https://www.alzint.org/resource/world-alzheimer-report-2022/.

[12] Burns A., Jacoby R., Luthert P., Levy R. (1990). Cause of Death in Alzheimer’s Disease. J. Am. Geriatr. Soc..

[13] Beard C., Kokmen E., Sigler C., Smith G.E., Petterson T., O’Brien P.C. (1996). Cause of death in Alzheimer’s disease. Ann. Epidemiol..

[14] Attems J., König C., Huber M., Lintner F., Jellinger K.A. (2005). Cause of death in demented and non-demented elderly inpatients; an autopsy study of 308 cases. J. Alzheimer’s Dis..

[15] Brunnström H.R., Englund E.M. (2009). Cause of death in patients with dementia disorders. Eur. J. Neurol..

[16] Todd S., Barr S., Passmore A.P. (2013). Cause of death in Alzheimer’s disease: A cohort study. QJM Int. J. Med..

[17] Romero J.P., Benito-León J., Louis E.D., Bermejo-Pareja F. (2014). Under Reporting of Dementia Deaths on Death Certificates: A Systematic Review of Population-based Cohort Studies. J. Alzheimer’s Dis..

[18] Foley N.C., Affoo R.H., Martin R.E. (2014). A Systematic Review and Meta-Analysis Examining Pneumonia-Associated Mortality in Dementia. Dement. Geriatr. Cogn. Disord..

[19] Manabe T., Fujikura Y., Mizukami K., Akatsu H., Kudo K. (2019). Pneumonia-associated death in patients with dementia: A systematic review and meta-analysis. PLoS ONE.

[20] Chouinard J. (2000). Dysphagia in Alzheimer disease: A review. J. Nutr. Health Aging.

[21] Kalia M. (2003). Dysphagia and aspiration pneumonia in patients with Alzheimer’s disease. Metabolism.

[22] Kai K., Hashimoto M., Amano K., Tanaka H., Fukuhara R., Ikeda M. (2015). Relationship between Eating Disturbance and Dementia Severity in Patients with Alzheimer’s Disease. PLoS ONE.

[23] Li C.-H., Hsieh S.-W., Huang P., Liu H.-Y., Chen C.-H., Hung C.-H. (2022). Pharmacological Management of Dysphagia in Patients with Alzheimer’s Disease: A Narrative Review. Curr. Alzheimer Res..

[24] Billinger S.A., Vidoni E.D., Honea R.A., Burns J.M. (2011). Cardiorespiratory Response to Exercise Testing in Individuals With Alzheimer’s Disease. Arch. Phys. Med. Rehabil..

[25] Sanches V.S., Santos F.M., Fernandes J.M., Santos M.L., Müller P.T., Christofoletti G. (2014). Neurodegenerative Disorders Increase Decline in Respiratory Muscle Strength in Older Adults. Respir. Care.

[26] Sachdev P., Anstey K., Parslow R., Wen W., Maller J., Kumar R., Christensen H., Jorm A. (2006). Pulmonary Function, Cognitive Impairment and Brain Atrophy in a Middle-Aged Community Sample. Dement. Geriatr. Cogn. Disord..

[27] Frenzel S., Bis J.C., Gudmundsson E.F., O’donnell A., Simino J., Yaqub A., Bartz T.M., Brusselle G.G.O., Bülow R., DeCarli C.S. (2022). Associations of Pulmonary Function with MRI Brain Volumes: A Coordinated Multi-Study Analysis. J. Alzheimer’s Dis..

[28] Russ T.C., Kivimäki M., Batty G.D. (2020). Respiratory Disease and Lower Pulmonary Function as Risk Factors for Dementia. Chest.

[29] Xiao T., Wijnant S.R., Licher S., Terzikhan N., Lahousse L., Ikram M.K., Brusselle G.G. (2021). Lung Function Impairment and the Risk of Incident Dementia: The Rotterdam Study. J. Alzheimer’s Dis..

[30] Hassel E., Stensvold D., Halvorsen T., Wisløff U., Langhammer A., Steinshamn S. (2015). Association between pulmonary function and peak oxygen uptake in elderly: The Generation 100 study. Respir. Res..

[31] Yaffe K., Laffan A.M., Harrison S.L., Redline S., Spira A.P., Ensrud K.E., Ancoli-Israel S., Stone K.L. (2011). Sleep-Disordered Breathing, Hypoxia, and Risk of Mild Cognitive Impairment and Dementia in Older Women. JAMA.

[32] Zhang F., Niu L., Li S., Le W. (2018). Pathological Impacts of Chronic Hypoxia on Alzheimer’s Disease. ACS Chem. Neurosci..

[33] Kaushal A., Wani W.Y., Bal A., Gill K.D., Kaur J. (2019). Okadaic Acid and Hypoxia Induced Dementia Model of Alzheimer’s Type in Rats. Neurotox. Res..

[34] Gaig C., Iranzo A. (2012). Sleep-Disordered Breathing in Neurodegenerative Diseases. Curr. Neurol. Neurosci. Rep..

[35] Mitchell S.L., Kiely D.K., Hamel M.B. (2004). Dying With Advanced Dementia in the Nursing Home. Arch. Intern. Med..

[36] Parvizi J., Van Hoesen G.W., Damasio A. (2001). The selective vulnerability of brainstem nuclei to Alzheimer’s disease. Ann. Neurol..

[37] Ehrenberg A.J., Nguy A.K., Theofilas P., Dunlop S., Suemoto C.K., Alho A.T.D.L., Leite R.P., Rodriguez R.D., Mejia M.B., Rüb U. (2017). Quantifying the accretion of hyperphosphorylated tau in the locus coeruleus and dorsal raphe nucleus: The pathological building blocks of early Alzheimer’s disease. Neuropathol. Appl. Neurobiol..

[38] Engelhardt E., Laks J. (2008). Alzheimer disease neuropathology:understanding autonomic dysfunction. Dement. Neuropsychol..

[39] Tulbă D., Cozma L., Popescu B.O., Davidescu E.I. (2020). Dysautonomia in Alzheimer’s Disease. Medicina.

[40] Lee J.H., Ryan J., Andreescu C., Aizenstein H., Lim H.K. (2015). Brainstem morphological changes in Alzheimer’s disease. NeuroReport.

[41] Sforza E., Roche F. (2016). Chronic intermittent hypoxia and obstructive sleep apnea: An experimental and clinical approach. Hypoxia.

[42] Snyder B., Shell B., Cunningham J.T., Cunningham R.L. (2017). Chronic intermittent hypoxia induces oxidative stress and inflammation in brain regions associated with early-stage neurodegeneration. Physiol. Rep..

[43] Merelli A., Repetto M., Lazarowski A., Auzmendi J. (2021). Hypoxia, Oxidative Stress, and Inflammation: Three Faces of Neurodegenerative Diseases. J. Alzheimer’s Dis..

[44] Dempsey J.A., Veasey S.C., Morgan B.J., O’Donnell C.P. (2010). Pathophysiology of Sleep Apnea. Physiol. Rev..

[45] Peppard P.E., Young T., Barnet J.H., Palta M., Hagen E.W., Hla K.M. (2013). Increased Prevalence of Sleep-Disordered Breathing in Adults. Am. J. Epidemiol..

[46] Dempsey J.A., Xie A., Patz D.S., Wang D. (2014). Physiology in Medicine: Obstructive sleep apnea pathogenesis and treatment—Considerations beyond airway anatomy. J. Appl. Physiol..

[47] Shepard J.W. (1992). Hypertension, cardiac arrhythmias, myocardial infarction, and stroke in relation to obstructive sleep apnea. Clin. Chest Med..

[48] Krysta K., Bratek A., Zawada K., Stepańczak R. (2017). Cognitive Deficits in Adults with Obstructive Sleep Apnea Compared to Children and Adolescents. J. Neural Transm..

[49] Patil S.P., Ayappa I.A., Caples S.M., Kimoff R.J., Patel S.R., Harrod C.G. (2019). Treatment of Adult Obstructive Sleep Apnea with Positive Airway Pressure: An American Academy of Sleep Medicine Clinical Practice Guideline. Sleep Med..

[50] Seda G., Han T.S. (2019). Effect of Obstructive Sleep Apnea on Neurocognitive Performance. Sleep Med. Clin..

[51] Lal C., Ayappa I., Ayas N., Beaudin A.E., Hoyos C., Kushida C.A., Kaminska M., Mullins A., Naismith S.L., Osorio R.S. (2022). The Link between Obstructive Sleep Apnea and Neurocognitive Impairment: An Official American Thoracic Society Workshop Report. Ann. Am. Thorac. Soc..

[52] Senaratna C.V., Perret J.L., Lodge C.J., Lowe A.J., Campbell B.E., Matheson M.C., Hamilton G.S., Dharmage S.C. (2017). Prevalence of obstructive sleep apnea in the general population: A systematic review. Sleep Med. Rev..

[53] Garvey J.F., Pengo M.F., Drakatos P., Kent B.D. (2015). Epidemiological aspects of obstructive sleep apnea. J. Thorac. Dis..

[54] Franklin K.A., Lindberg E. (2015). Obstructive sleep apnea is a common disorder in the population—A review on the epidemiology of sleep apnea. J. Thorac. Dis..

[55] Heinzer R., Vat S., Marques-Vidal P., Marti-Soler H., Andries D., Tobback N., Mooser V., Preisig M., Malhotra A., Waeber G. (2015). Prevalence of sleep-disordered breathing in the general population: The HypnoLaus study. Lancet Respir. Med..

[56] Hongyo K., Ito N., Yamamoto K., Yasunobe Y., Takeda M., Oguro R., Takami Y., Takeya Y., Sugimoto K., Rakugi H. (2016). Factors associated with the severity of obstructive sleep apnea in older adults. Geriatr. Gerontol. Int..

[57] Marde V.S., Atkare U.A., Gawali S.V., Tiwari P.L., Badole S.P., Wankhede N.L., Taksande B.G., Upaganlawar A.B., Umekar M.J., Kale M.B. (2021). Alzheimer’s disease and sleep disorders: Insights into the possible disease connections and the potential therapeutic targets. Asian J. Psychiatry.

[58] Hirano A., Zimmerman H.M. (1962). Alzheimer’s Neurofibrillary Changes. Arch. Neurol..

[59] Lloret M.-A., Cervera-Ferri A., Nepomuceno M., Monllor P., Esteve D., Lloret A. (2020). Is Sleep Disruption a Cause or Consequence of Alzheimer’s Disease? Reviewing Its Possible Role as a Biomarker. Int. J. Mol. Sci..

[60] Reynolds C.F., Kupfer D.J., Taska L.S., Hoch C.C., E Sewitch D., Restifo K., Spiker D.G., Zimmer B., Marin R.S., Nelson J. (1985). Sleep apnea in Alzheimer’s dementia: Correlation with mental deterioration. J. Clin. Psychiatry.

[61] Erkinjuntti T., Partinen M., Sulkava R., Telakivi T., Salmi T., Tilvis R. (1987). Sleep Apnea in Multiinfarct Dementia and Alzheimer’s Disease. Sleep.

[62] Eemamian F., Ekhazaie H., Tahmasian M., Leschziner G.D., Morrell M.J., Hsiung G.-Y.R., Erosenzweig I., Sepehry A. (2016). The Association Between Obstructive Sleep Apnea and Alzheimer’s Disease: A Meta-Analysis Perspective. Front. Aging Neurosci..

[63] Andrade A.G., Bubu O.M., Varga A.W., Osorio R.S. (2018). The Relationship between Obstructive Sleep Apnea and Alzheimer’s Disease. J. Alzheimer’s Dis..

[64] Gosselin N., Baril A.-A., Osorio R.S., Kaminska M., Carrier J. (2019). Obstructive Sleep Apnea and the Risk of Cognitive Decline in Older Adults. Am. J. Respir. Crit. Care Med..

[65] Bubu O.M., Andrade A.G., Umasabor-Bubu O.Q., Hogan M.M., Turner A.D., de Leon M.J., Ogedegbe G., Ayappa I., Jackson M.L., Varga A.W. (2019). Obstructive sleep apnea, cognition and Alzheimer’s disease: A systematic review integrating three decades of multidisciplinary research. Sleep Med. Rev..

[66] Leng Y., McEvoy C.T., Allen I.E., Yaffe K. (2017). Association of Sleep-Disordered Breathing With Cognitive Function and Risk of Cognitive Impairment. JAMA Neurol..

[67] Zhu X., Zhao Y. (2017). Sleep-disordered breathing and the risk of cognitive decline: A meta-analysis of 19,940 participants. Sleep Breath..

[68] Osorio R.S., Gumb T., Pirraglia E., Varga A.W., Lu S.-E., Lim J., Wohlleber M.E., Ducca E.L., Koushyk V., Glodzik L. (2015). Sleep-disordered breathing advances cognitive decline in the elderly. Neurology.

[69] Benedict C., Byberg L., Cedernaes J., Hogenkamp P.S., Giedratis V., Kilander L., Lind L., Lannfelt L., Schiöth H.B. (2014). Self-reported sleep disturbance is associated with Alzheimer’s disease risk in men. Alzheimer’s Dement..

[70] Lee J.E., Yang S.W., Ju Y.J., Ki S.K., Chun K.H. (2019). Sleep-disordered breathing and Alzheimer’s disease: A nationwide cohort study. Psychiatry Res..

[71] André C., Rehel S., Kuhn E., Landeau B., Moulinet I., Touron E., Ourry V., Le Du G., Mézenge F., Tomadesso C. (2020). Association of Sleep-Disordered Breathing With Alzheimer Disease Biomarkers in Community-Dwelling Older Adults. JAMA Neurol..

[72] Cooke J.R., Ayalon L., Palmer B.W., Loredo J.S., Corey-Bloom J., Natarajan L., Liu L., Ancoli-Israel S. (2009). Sustained Use of CPAP Slows Deterioration of Cognition, Sleep, and Mood in Patients with Alzheimer’s Disease and Obstructive Sleep Apnea: A Preliminary Study. Sleep Med..

[73] Richards K.C., Gooneratne N., Dicicco B., Hanlon A., Moelter S., Onen F., Wang Y., Sawyer A., Weaver T., Lozano A. (2019). CPAP Adherence May Slow 1-Year Cognitive Decline in Older Adults with Mild Cognitive Impairment and Apnea. J. Am. Geriatr. Soc..

[74] Wang Y., Cheng C., Moelter S., Fuentecilla J.L., Kincheloe K., Lozano A.J., Carter P., Gooneratne N., Richards K.C. (2020). One Year of Continuous Positive Airway Pressure Adherence Improves Cognition in Older Adults With Mild Apnea and Mild Cognitive Impairment. Nurs. Res..

[75] Liguori C., Cremascoli R., Maestri M., Fernandes M., Izzi F., Tognoni G., Scarpina F., Siciliano G., Mercuri N.B., Priano L. (2021). Obstructive Sleep Apnea Syndrome and Alzheimer’s Disease Pathology: May Continuous Positive Airway Pressure Treatment Delay Cognitive Deterioration?. Sleep Breath..

[76] Jiang X., Wang Z., Hu N., Yang Y., Xiong R., Fu Z. (2021). Cognition effectiveness of continuous positive airway pressure treatment in obstructive sleep apnea syndrome patients with cognitive impairment: A meta-analysis. Exp. Brain Res..

[77] Shieu M.M., Zaheed A.B., Shannon C., Chervin R.D., Conceicao A., Paulson H.L., Braley T.J., Dunietz G.L. (2022). Positive Airway Pressure and Cognitive Disorders in Adults With Obstructive Sleep Apnea. Neurology.

[78] Dunietz G.L., Chervin R.D., Burke J.F., Conceicao A.S., Braley T.J. (2021). Obstructive sleep apnea treatment and dementia risk in older adults. Sleep.

[79] Fernandes M., Placidi F., Mercuri N.B., Liguori C. (2021). The Importance of Diagnosing and the Clinical Potential of Treating Obstructive Sleep Apnea to Delay Mild Cognitive Impairment and Alzheimer’s Disease: A Special Focus on Cognitive Performance. J. Alzheimer’s Dis. Rep..

[80] DeVettori G., Troxel W.M., Duff K., Baron K.G. (2023). Positive airway pressure adherence among patients with obstructive sleep apnea and cognitive impairment: A narrative review. Sleep Med..

[81] Lajoie A.C., Gu Y., Lim A., Benedetti A., Kaminska M. (2023). Adherence to continuous positive airway pressure for the treatment of obstructive sleep apnea in neurodegenerative diseases: A systematic review. Sleep Med. Rev..

[82] Polsek D., Gildeh N., Cash D., Winsky-Sommerer R., Williams S., Turkheimer F., Leschziner G., Morrell M., Rosenzweig I. (2017). Obstructive sleep apnoea and Alzheimer’s disease: In search of shared pathomechanisms. Neurosci. Biobehav. Rev..

[83] D’Rozario A.L., Field C.J., Hoyos C.M., Naismith S.L., Dungan G.C., Wong K.K.H., Grunstein R.R., Bartlett D.J. (2018). Impaired Neurobehavioural Performance in Untreated Obstructive Sleep Apnea Patients Using a Novel Standardised Test Battery. Front. Surg..

[84] Beaudin A.E., Raneri J.K., Ayas N.T., Skomro R.P., Fox N., Allen A.J.M.H., Bowen M.W., Nocon A., Lynch E.J., Wang M. (2021). Cognitive Function in a Sleep Clinic Cohort of Patients with Obstructive Sleep Apnea. Ann. Am. Thorac. Soc..

[85] Weihs A., Frenzel S., Grabe H.J. (2021). The Link Between Obstructive Sleep Apnoea and Neurodegeneration and Cognition. Curr. Sleep Med. Rep..

[86] Gao F., Wei S., Dang L., Gao Y., Gao L., Shang S., Chen C., Huo K., Wang J., Wang J. (2022). Sleep disturbance is associated with mild cognitive impairment: A community population-based cross-sectional study. BMC Public Health.

[87] Chien W.-C., Lin C.-W., Liu C.-K., Chen S.-L., Chou M.-C., Hsu C.-Y. (2023). The Associations between Polysomnographic Parameters and Memory Impairment among Patients with Obstructive Sleep Apnea: A 10-Year Hospital-Based Longitudinal Study. Biomedicines.

[88] Hanslik K.L., Ulland T.K. (2020). The Role of Microglia and the Nlrp3 Inflammasome in Alzheimer’s Disease. Front. Neurol..

[89] Kheirandish-Gozal L., Gozal D. (2019). Obstructive Sleep Apnea and Inflammation: Proof of Concept Based on Two Illustrative Cytokines. Int. J. Mol. Sci..

[90] Liu X., Ma Y., Ouyang R., Zeng Z., Zhan Z., Lu H., Cui Y., Dai Z., Luo L., He C. (2020). The relationship between inflammation and neurocognitive dysfunction in obstructive sleep apnea syndrome. J. Neuroinflammation.

[91] Gomez-Nicola D., Boche D. (2015). Post-mortem analysis of neuroinflammatory changes in human Alzheimer’s disease. Alzheimer’s Res. Ther..

[92] Hamelin L., Lagarde J., Dorothée G., Leroy C., Labit M., Comley R.A., de Souza L.C., Corne H., Dauphinot L., Bertoux M. (2016). Early and protective microglial activation in Alzheimer’s disease: A prospective study using18F-DPA-714 PET imaging. Brain.

[93] Knezevic D., Mizrahi R. (2018). Molecular imaging of neuroinflammation in Alzheimer’s disease and mild cognitive impairment. Prog. Neuro-Psychopharmacol. Biol. Psychiatry.

[94] Bradburn S., Murgatroyd C., Ray N. (2019). Neuroinflammation in mild cognitive impairment and Alzheimer’s disease: A meta-analysis. Ageing Res. Rev..

[95] Kinney J.W., Bemiller S.M., Murtishaw A.S., Leisgang A.M., Salazar A.M., Lamb B.T. (2018). Inflammation as a central mechanism in Alzheimer’s disease. Alzheimer’s Dement. Transl. Res. Clin. Interv..

[96] Novoa C., Salazar P., Cisternas P., Gherardelli C., Vera-Salazar R., Zolezzi J.M., Inestrosa N.C. (2022). Inflammation context in Alzheimer’s disease, a relationship intricate to define. Biol. Res..

[97] Liu G., Yang C., Wang X., Chen X., Wang Y., Le W. (2023). Oxygen metabolism abnormality and Alzheimer’s disease: An update. Redox Biol..

[98] Zabel M., Nackenoff A., Kirsch W.M., Harrison F.E., Perry G., Schrag M. (2017). Markers of oxidative damage to lipids, nucleic acids and proteins and antioxidant enzymes activities in Alzheimer’s disease brain: A meta-analysis in human pathological specimens. Free Radic. Biol. Med..

[99] Cassidy L., Fernandez F., Johnson J.B., Naiker M., Owoola A.G., Broszczak D.A. (2019). Oxidative stress in alzheimer’s disease: A review on emergent natural polyphenolic therapeutics. Complement. Ther. Med..

[100] Kowalczyk P., Sulejczak D., Kleczkowska P., Bukowska-Ośko I., Kucia M., Popiel M., Wietrak E., Kramkowski K., Wrzosek K., Kaczyńska K. (2021). Mitochondrial Oxidative Stress—A Causative Factor and Therapeutic Target in Many Diseases. Int. J. Mol. Sci..

[101] Song T., Song X., Zhu C., Patrick R., Skurla M., Santangelo I., Green M., Harper D., Ren B., Forester B.P. (2021). Mitochondrial dysfunction, oxidative stress, neuroinflammation, and metabolic alterations in the progression of Alzheimer’s disease: A meta-analysis of in vivo magnetic resonance spectroscopy studies. Ageing Res. Rev..

[102] Huang W.J., Zhang X., Chen W.W. (2016). Role of oxidative stress in Alzheimer’s disease. Biomed. Rep..

[103] Owen J.E., Benediktsdottir B., Cook E., Olafsson I., Gislason T., Robinson S.R. (2021). Alzheimer’s Disease Neuropathology in the Hippocampus and Brainstem of People with Obstructive Sleep Apnea. Sleep.

[104] Zhang C.-E., Yang X., Li L., Sui X., Tian Q., Wei W., Wang J., Liu G. (2014). Hypoxia-Induced Tau Phosphorylation and Memory Deficit in Rats. Neurodegener. Dis..

[105] Yagishita S., Suzuki S., Yoshikawa K., Iida K., Hirata A., Suzuki M., Takashima A., Maruyama K., Hirasawa A., Awaji T. (2017). Treatment of intermittent hypoxia increases phosphorylated tau in the hippocampus via biological processes common to aging. Mol. Brain.

[106] Kazim S.F., Sharma A., Saroja S.R., Seo J.H., Larson C.S., Ramakrishnan A., Wang M., Blitzer R.D., Shen L., Peña C.J. (2021). Chronic Intermittent Hypoxia Enhances Pathological Tau Seeding, Propagation, and Accumulation and Exacerbates Alzheimer-like Memory and Synaptic Plasticity Deficits and Molecular Signatures. Biol. Psychiatry.

[107] Lei L., Feng J., Wu G., Wei Z., Wang J.Z., Zhang B., Liu R., Liu F., Wang X., Li H.L. (2022). HIF-1α Causes LCMT1/PP2A Deficiency and Mediates Tau Hyperphosphorylation and Cognitive Dysfunction during Chronic Hypoxia. Int. J. Mol. Sci..

[108] Gozal D., Row B.W., Kheirandish L., Liu R., Guo S.Z., Qiang F., Brittian K.R. (2003). Increased susceptibility to intermittent hypoxia in aging rats: Changes in proteasomal activity, neuronal apoptosis and spatial function. J. Neurochem..

[109] Xu W., Chi L., Row B.W., Xu R., Ke Y., Xu B., Luo C., Kheirandish L., Gozal D., Liu R. (2004). Increased oxidative stress is associated with chronic intermittent hypoxia-mediated brain cortical neuronal cell apoptosis in a mouse model of sleep apnea. Neuroscience.

[110] Reitz C., Mayeux R. (2014). Alzheimer disease: Epidemiology, diagnostic criteria, risk factors and biomarkers. Biochem. Pharmacol..

[111] Planche V., Manjon J.V., Mansencal B., Lanuza E., Tourdias T., Catheline G., Coupé P. (2022). Structural progression of Alzheimer’s disease over decades: The MRI staging scheme. Brain Commun..

[112] Morrell M.J., Jackson M.L., Twigg G.L., Ghiassi R., McRobbie D.W., Quest R.A., Pardoe H., Pell G.S., Abbott D.F., Rochford P.D. (2010). Changes in brain morphology in patients with obstructive sleep apnoea. Thorax.

[113] Canessa N., Castronovo V., Cappa S.F., Aloia M.S., Marelli S., Falini A., Alemanno F., Ferini-Strambi L. (2011). Obstructive Sleep Apnea: Brain Structural Changes and Neurocognitive Function before and after Treatment. Am. J. Respir. Crit. Care Med..

[114] Joo E.Y., Jeon S., Kim S.T., Lee J.-M., Hong S.B. (2013). Localized Cortical Thinning in Patients with Obstructive Sleep Apnea Syndrome. Sleep.

[115] Kim R.E.Y., Abbott R.D., Kim S., Thomas R.J., Yun C.-H., Kim H., Johnson H., Shin C. (2021). Sleep Duration, Sleep Apnea, and Gray Matter Volume. J. Geriatr. Psychiatry Neurol..

[116] Owen J.E., Benediktsdóttir B., Gislason T., Robinson S.R. (2019). Neuropathological investigation of cell layer thickness and myelination in the hippocampus of people with obstructive sleep apnea. Sleep.

[117] Chokesuwattanaskul A., Chirakalwasan N., Jaimchariyatam N., Pitakvej N., Sarutikriangkri Y., Chunharas C., Phanthumchinda K., Likitjaroen Y. (2020). Associations between hypoxia parameters in obstructive sleep apnea and cognition, cortical thickness, and white matter integrity in middle-aged and older adults. Sleep Breath..

[118] Díaz-Román M., Pulopulos M.M., Baquero M., Salvador A., Cuevas A., Ferrer I., Ciopat O., Gómez E. (2020). Obstructive sleep apnea and Alzheimer’s disease-related cerebrospinal fluid biomarkers in mild cognitive impairment. Sleep.

[119] Przybylska-Kuć S., Zakrzewski M., Dybała A., Kiciński P., Dzida G., Myśliński W., Prystupa A., Mosiewicz-Madejska B., Mosiewicz J. (2019). Obstructive sleep apnea may increase the risk of Alzheimer’s disease. PLoS ONE.

[120] Kong W., Zheng Y., Xu W., Gu H., Wu J. (2020). Biomarkers of Alzheimer’s disease in severe obstructive sleep apnea–hypopnea syndrome in the Chinese population. Eur. Arch. Oto-Rhino-Laryngol..

[121] Kang J., Tian Z., Wei J., Mu Z., Liang J., Li M. (2022). Association between obstructive sleep apnea and Alzheimer’s disease-related blood and cerebrospinal fluid biomarkers: A meta-analysis. J. Clin. Neurosci..

[122] Huang Z., Zeng H., Huang Y., Wang T., Huang W., Huang Y., Lin L., Li H. (2023). The relationship between obstructive sleep apnea and circulating tau levels: A meta-analysis. Brain Behav..

[123] Ju Y.-E.S., Finn M.B., Sutphen C.L., Herries E.M., Jerome G.M., Ladenson J.H., Crimmins D.L., Fagan A.M., Holtzman D.M. (2016). Obstructive sleep apnea decreases central nervous system-derived proteins in the cerebrospinal fluid. Ann. Neurol..

[124] Harrison I.F., Ismail O., Machhada A., Colgan N., Ohene Y., Nahavandi P., Ahmed Z., Fisher A., Meftah S., Murray T.K. (2020). Impaired glymphatic function and clearance of tau in an Alzheimer’s disease model. Brain.

[125] Silva I., Silva J., Ferreira R., Trigo D. (2021). Glymphatic System, AQP4, and Their Implications in Alzheimer’s Disease. Neurol. Res. Pract..

[126] Roy B., Nunez A., Aysola R.S., Kang D.W., Vacas S., Kumar R. (2022). Impaired Glymphatic System Actions in Obstructive Sleep Apnea Adults. Front. Neurosci..

[127] Wang J., Tian Y., Qin C., Meng L., Feng R., Xu S., Zhai Y., Liang D., Zhang R., Tian H. (2023). Impaired glymphatic drainage underlying obstructive sleep apnea is associated with cognitive dysfunction. J. Neurol..

[128] Dakterzada F., Benítez I.D., Targa A., Carnes A., Pujol M., Jové M., Mínguez O., Vaca R., Sánchez-De-La-Torre M., Barbé F. (2023). Cerebrospinal fluid lipidomic fingerprint of obstructive sleep apnoea in Alzheimer’s disease. Alzheimer’s Res. Ther..

[129] Chew H., Solomon V.A., Fonteh A.N. (2020). Involvement of Lipids in Alzheimer’s Disease Pathology and Potential Therapies. Front. Physiol..

[130] Gharbi-Meliani A., Dugravot A., Sabia S., Regy M., Fayosse A., Schnitzler A., Kivimäki M., Singh-Manoux A., Dumurgier J. (2021). The association of APOE ε4 with cognitive function over the adult life course and incidence of dementia: 20 years follow-up of the Whitehall II study. Alzheimer’s Res. Ther..

[131] Sienski G., Narayan P., Bonner J.M., Kory N., Boland S., Arczewska A.A., Ralvenius W.T., Akay L., Lockshin E., He L. (2021). APOE4 Disrupts Intracellular Lipid Homeostasis in Human IPSC-Derived Glia. Sci. Transl. Med..

[132] Raulin A.-C., Doss S.V., Trottier Z.A., Ikezu T.C., Bu G., Liu C.-C. (2022). ApoE in Alzheimer’s disease: Pathophysiology and therapeutic strategies. Mol. Neurodegener..

[133] Costa-Laparra I., Juárez-Escoto E., Vicario C., Moratalla R., García-Sanz P. (2023). APOE Ε4 Allele, along with G206D-PSEN1 Mutation, Alters Mitochondrial Networks and Their Degradation in Alzheimer’s Disease. Front. Aging Neurosci..

[134] Blackman J., Love S., Sinclair L., Cain R., Coulthard E. (2022). APOE Ε4, Alzheimer’s Disease Neuropathology and Sleep Disturbance, in Individuals with and without Dementia. Alzheimer’s Res. Ther..

[135] Wei W., Wang K., Shi J., Li Z. (2022). The Relationship Between Sleep Disturbance and Apolipoprotein E ε4 in Adults With Mild Cognitive Impairment and Alzheimer’s Disease Dementia: An Integrative Review. Biol. Res. Nurs..

[136] Gottlieb D.J., DeStefano A.L., Foley D.J., Mignot E., Redline S., Givelber R.J., Young T. (2004). APOE ε4 is associated with obstructive sleep apnea/hypopnea. Neurology.

[137] Uyrum E., Balbay O., Annakkaya A.N., Balbay E.G., Silan F., Arbak P. (2015). The Relationship between Obstructive Sleep Apnea Syndrome and Apolipoprotein E Genetic Variants. Respiration.

[138] Thakre T.P., Mamtani M.R., Kulkarni H. (2009). Lack of Association of the APOE ε4 Allele with the Risk of Obstructive Sleep Apnea: Meta-Analysis and Meta-Regression. Sleep.

[139] Xu H., Qian Y., Guan J., Yi H., Yin S. (2015). No association between the ApoE ε2 and ε4 alleles and the risk of obstructive sleep apnea: A systematic review and meta-analysis. Biomed. Rep..

[140] Research Models|ALZFORUM. https://www.alzforum.org/research-models.

[141] Drummond E., Wisniewski T. (2016). Alzheimer’s disease: Experimental models and reality. Acta Neuropathol..

[142] Götz J., Bodea L.-G., Goedert M. (2018). Rodent models for Alzheimer disease. Nat. Rev. Neurosci..

[143] Ebel D.L., Torkilsen C.G., Ostrowski T.D. (2017). Blunted Respiratory Responses in the Streptozotocin-Induced Alzheimer’s Disease Rat Model. J. Alzheimer’s Dis..

[144] Vicente M.C., Humphrey C.M., Gargaglioni L.H., Ostrowski T.D. (2020). Decreased excitability of locus coeruleus neurons during hypercapnia is exaggerated in the streptozotocin-model of Alzheimer’s disease. Exp. Neurol..

[145] Brown A.G., Thapa M., Hooker J.W., Ostrowski T.D. (2018). Impaired chemoreflex correlates with decreased c-Fos in respiratory brainstem centers of the streptozotocin-induced Alzheimer’s disease rat model. Exp. Neurol..

[146] Humphrey C.M., Hooker J.W., Thapa M., Wilcox M.J., Ostrowski D., Ostrowski T.D. (2023). Synaptic loss and gliosis in the nucleus tractus solitarii with streptozotocin-induced Alzheimer’s disease. Brain Res..

[147] Kamat P.K. (2015). Streptozotocin induced Alzheimer’s disease like changes and the underlying neural degeneration and regeneration mechanism. Neural Regen. Res..

[148] Grieb P. (2015). Intracerebroventricular Streptozotocin Injections as a Model of Alzheimer’s Disease: In Search of a Relevant Mechanism. Mol. Neurobiol..

[149] Salkovic-Petrisic M., Hoyer S. (2007). Central Insulin Resistance as a Trigger for Sporadic Alzheimer-like Pathology: An Experimental Approach. J. Neural. Transm. Suppl..

[150] Rai S., Kamat P.K., Nath C., Shukla R. (2014). Glial activation and post-synaptic neurotoxicity: The key events in Streptozotocin (ICV) induced memory impairment in rats. Pharmacol. Biochem. Behav..

[151] Ravelli K.G., Rosário B.d.A., Camarini R., Hernandes M.S., Britto L.R. (2016). Intracerebroventricular Streptozotocin as a Model of Alzheimer’s Disease: Neurochemical and Behavioral Characterization in Mice. Neurotox. Res..

[152] Kraska A., Santin M.D., Dorieux O., Joseph-Mathurin N., Bourrin E., Petit F., Jan C., Chaigneau M., Hantraye P., Lestage P. (2012). In Vivo Cross-sectional Characterization of Cerebral Alterations Induced by Intracerebroventricular Administration of Streptozotocin. PLoS ONE.

[153] Vicente M.C., Almeida M.C., Bícego K.C., Carrettiero D.C., Gargaglioni L.H. (2018). Hypercapnic and Hypoxic Respiratory Response During Wakefulness and Sleep in a Streptozotocin Model of Alzheimer’s Disease in Rats. J. Alzheimer’s Dis..

[154] Vicente M.C., Paneghini J.L., Stabile A.M., Amorim M., Silva C.E.A., Patrone L.G.A., Cunha T.M., Bícego K.C., Almeida M.C., Carrettiero D.C. (2023). Inhibition of Pro-Inflammatory Microglia with Minocycline Improves Cognitive and Sleep-Wake Dysfunction Under Respiratory Stress in a Sporadic Model for Alzheimer’s Disease. J. Alzheimer’s Dis..

[155] Biancardi V., Bícego K.C., Almeida M.C., Gargaglioni L.H. (2007). Locus coeruleus noradrenergic neurons and CO_2_ drive to breathing. Pflügers Arch. Eur. J. Physiol..

[156] Benarroch E.E. (2009). The locus ceruleus norepinephrine system. Neurology.

[157] Magalhães K.S., Spiller P.F., da Silva M.P., Kuntze L.B., Paton J.F.R., Machado B.H., Moraes D.J.A. (2018). Locus Coeruleus as a vigilance centre for active inspiration and expiration in rats. Sci. Rep..

[158] Jordan D. (1994). Central Integration of Chemoreceptor Afferent Activity. Adv. Exp. Med. Biol..

[159] Sapru H.N. (1996). Carotid chemoreflex. Neural Pathw. Transm..

[160] Zoccal D.B., Furuya W.I., Bassi M., Colombari D.S.A., Colombari E. (2014). The nucleus of the solitary tract and the coordination of respiratory and sympathetic activities. Front. Physiol..

[161] Shonesy B.C., Thiruchelvam K., Parameshwaran K., Rahman E.A., Karuppagounder S.S., Huggins K.W., Pinkert C.A., Amin R., Dhanasekaran M., Suppiramaniam V. (2011). Central insulin resistance and synaptic dysfunction in intracerebroventricular-streptozotocin injected rodents. Neurobiol. Aging.

[162] Bazilio D.S., Rodrigues K.L., Moraes D.J., Machado B.H. (2020). Distinct cardiovascular and respiratory responses to short-term sustained hypoxia in juvenile Sprague Dawley and Wistar Hannover rats. Auton. Neurosci..

[163] Putnam R.W., Filosa J.A., Ritucci N.A. (2004). Cellular mechanisms involved in CO_2_ and acid signaling in chemosensitive neurons. Am. J. Physiol. Physiol..

[164] Nestor S.M., Rupsingh R., Borrie M., Smith M., Accomazzi V., Wells J.L., Fogarty J., Bartha R., Initiative T.A.D.N. (2008). Ventricular enlargement as a possible measure of Alzheimer’s disease progression validated using the Alzheimer’s disease neuroimaging initiative database. Brain.

[165] Apostolova L.G.M., Green A.E.B., Babakchanian S.B., Hwang K.S.B., Chou Y.-Y., Toga A.W., Thompson P.M. (2012). Hippocampal Atrophy and Ventricular Enlargement in Normal Aging, Mild Cognitive Impairment (MCI), and Alzheimer Disease. Alzheimer Dis. Assoc. Disord..

[166] Gannon M., Che P., Chen Y., Jiao K., Roberson E.D., Wang Q. (2015). Noradrenergic dysfunction in Alzheimer’s disease. Front. Neurosci..

[167] Peterson A.C., Li C.-S.R. (2018). Noradrenergic Dysfunction in Alzheimer’s and Parkinson’s Diseases—An Overview of Imaging Studies. Front. Aging Neurosci..

[168] Eser R.A., Ehrenberg A.J., Petersen C., Dunlop S., Mejia M.B., Suemoto C.K., Walsh C.M., Rajana H., Oh J., Theofilas P. (2018). Selective Vulnerability of Brainstem Nuclei in Distinct Tauopathies: A Postmortem Study. J. Neuropathol. Exp. Neurol..

[169] Simic G., Stanic G., Mladinov M., Jovanov-Milosevic N., Kostovic I., Hof P.R. (2009). Does Alzheimer’s disease begin in the brainstem?. Neuropathol. Appl. Neurobiol..

[170] Daulatzai M.A. (2012). Dysfunctional Nucleus Tractus Solitarius: Its Crucial Role in Promoting Neuropathogentic Cascade of Alzheimer’s Dementia—A Novel Hypothesis. Neurochem. Res..

[171] Brzecka A., Leszek J., Ashraf G.M., Ejma M., Ávila-Rodriguez M.F., Yarla N.S., Tarasov V.V., Chubarev V.N., Samsonova A.N., Barreto G.E. (2018). Sleep Disorders Associated With Alzheimer’s Disease: A Perspective. Front. Neurosci..

[172] Cohen R.M., Rezai-Zadeh K., Weitz T.M., Rentsendorj A., Gate D., Spivak I., Bholat Y., Vasilevko V., Glabe C.G., Breunig J.J. (2013). A Transgenic Alzheimer Rat with Plaques, Tau Pathology, Behavioral Impairment, Oligomeric Aβ, and Frank Neuronal Loss. J. Neurosci..

[173] Lucking E.F., Murphy K.H., Burns D.P., Jaisimha A.V., Barry-Murphy K.J., Dhaliwal P., Boland B., Rae M.G., O’halloran K.D. (2019). No evidence in support of a prodromal respiratory control signature in the TgF344-AD rat model of Alzheimer’s disease. Respir. Physiol. Neurobiol..

[174] Terwel D., Lasrado R., Snauwaert J., Vandeweert E., Van Haesendonck C., Borghgraef P., Van Leuven F. (2005). Changed Conformation of Mutant Tau-P301L Underlies the Moribund Tauopathy, Absent in Progressive, Nonlethal Axonopathy of Tau-4R/2N Transgenic Mice. J. Biol. Chem..

[175] Dutschmann M., Menuet C., Stettner G.M., Gestreau C., Borghgraef P., Devijver H., Gielis L., Hilaire G., Van Leuven F. (2010). Upper Airway Dysfunction of Tau-P301L Mice Correlates with Tauopathy in Midbrain and Ponto-Medullary Brainstem Nuclei. J. Neurosci..

[176] Menuet C., Borghgraef P., Matarazzo V., Gielis L., Lajard A.-M., Voituron N., Gestreau C., Dutschmann M., Van Leuven F., Hilaire G. (2011). Raphé tauopathy alters serotonin metabolism and breathing activity in terminal Tau.P301L mice: Possible implications for tauopathies and Alzheimer’s disease. Respir. Physiol. Neurobiol..

[177] Jęśko H., Wencel P.L., Lukiw W.J., Strosznajder R.P. (2018). Modulatory Effects of Fingolimod (FTY720) on the Expression of Sphingolipid Metabolism-Related Genes in an Animal Model of Alzheimer’s Disease. Mol. Neurobiol..

[178] Moechars D., Dewachter I., Lorent K., Reversé D., Baekelandt V., Naidu A., Tesseur I., Spittaels K., Haute C.V.D., Checler F. (1999). Early Phenotypic Changes in Transgenic Mice That Overexpress Different Mutants of Amyloid Precursor Protein in Brain. J. Biol. Chem..

[179] Howlett D.R., Richardson J.C. (2009). The pathology of APP transgenic mice: A model of Alzheimer’s disease or simply overexpression of APP?. Histol. Histopathol..

[180] Bronfman F.C., Moechars D., Van Leuven F. (2000). Acetylcholinesterase-Positive Fiber Deafferentation and Cell Shrinkage in the Septohippocampal Pathway of Aged Amyloid Precursor Protein London Mutant Transgenic Mice. Neurobiol. Dis..

[181] Andrzejewski K., Jampolska M., Mojzych I., Conde S.V., Kaczyńska K. (2022). Hypoxic and Hypercapnic Responses in Transgenic Murine Model of Alzheimer’s Disease Overexpressing Human AβPP: The Effects of Pretreatment with Memantine and Rivastigmine. Int. J. Mol. Sci..

[182] Bukke V.N., Archana M., Villani R., Romano A.D., Wawrzyniak A., Balawender K., Orkisz S., Beggiato S., Serviddio G., Cassano T. (2020). The Dual Role of Glutamatergic Neurotransmission in Alzheimer’s Disease: From Pathophysiology to Pharmacotherapy. Int. J. Mol. Sci..

